# PD50 Certainty Of Evidence Assessment Of The BRIGHTE Study: Fostemsavir In The Treatment Of Multidrug-Resistant HIV-1

**DOI:** 10.1017/S0266462325101827

**Published:** 2025-12-29

**Authors:** Marina Silva, Isabela Rossi, Lívia Nassif, Roberto Júnior, Ludmila Gargano, Juliana Alvares-Teodoro, Francisco Acurcio, Augusto Guerra Júnior

## Abstract

**Introduction:**

The BRIGHTE study assessed the efficacy of fostemsavir tromethamine 600 mg with optimized background therapy in adults with multidrug-resistant HIV-1 and virological failure. Participants were divided into a randomized cohort, comparing fostemsavir with placebo, and an observational cohort. After eight days, all randomized participants switched to fostemsavir. This study evaluated the certainty of evidence from the BRIGHTE trial.

**Methods:**

The GRADEpro tool was used to assess the quality of evidence. Five studies related to BRIGHTE were included, covering follow-up assessments at eight days (randomized phase) and 48, 96, and 240 weeks (observational phase). The outcomes assessed included changes in viral load, adverse events, virological response and failure, changes in CD4+ T cell count from baseline, mortality, quality of life, and treatment adherence. Differences between the randomized and observational phases were evaluated, focusing on the impact of this transition on evidence quality.

**Results:**

Transition to the observational phase compromised methodological robustness, increasing the risk of bias. In the randomized phase, evidence quality was reduced by the lack of details on the randomization process and uncertainties regarding double blinding. In the observational phase, confounding bias arose mainly due to intervention classification and the absence of an active comparator, hampering the assessment of fostemsavir’s relative efficacy. Intervention status influenced outcomes. Despite these limitations, fostemsavir showed consistent virological responses, indicating potential benefit for highly treatment-experienced individuals. Overall, evidence certainty was rated low for most outcomes, reflecting the combined impact of bias risks and methodological limitations.

**Conclusions:**

Although findings from the BRIGHTE study highlight fostemsavir’s clinical potential, the transition from a randomized controlled trial to an observational study reduced its evidence quality. The lack of comparators in the observational phase limited interpretations. However, a single-arm design is ethically justified for individuals with limited options to ensure access to interventions. Future studies should prioritize hybrid approaches and real-world data to improve clinical applicability.

